# *In vivo* localizations of membrane stress controllers PspA and PspG in *Escherichia coli*

**DOI:** 10.1111/j.1365-2958.2009.06776.x

**Published:** 2009-06-30

**Authors:** Christoph Engl, Goran Jovanovic, Louise J Lloyd, Heath Murray, Martin Spitaler, Liming Ying, Jeff Errington, Martin Buck

**Affiliations:** 1 Division of Biology, Sir Alexander Fleming Building, Imperial College LondonLondon SW7 2AZ, UK; 2 Institute for Cell and Molecular Biosciences, Catherine Cookson Building, University of NewcastleNewcastle NE2 4HH, UK; 3 FILM, Sir Alexander Fleming Building, Imperial College LondonLondon SW7 2AZ, UK; 4 Molecular Medicine, National Heart and Lung Institute, Imperial College LondonLondon SW7 2AZ, UK

## Abstract

The phage shock protein (Psp) response in Gram-negative bacteria counteracts membrane stress. Transcription of the PspF regulon (*pspABCDE* and *pspG*) in *Escherichia coli* is induced upon stresses that dissipate the proton motive force (pmf). Using GFP fusions we have visualized the subcellular localizations of PspA (a negative regulator and effector of Psp) and PspG (an effector of Psp). It has previously been proposed that PspA evenly coates the inner membrane of the cell. We now demonstrate that instead of uniformly covering the entire cell, PspA (and PspG) is highly organized into what appear to be distinct functional classes (complexes at the cell pole and the lateral cell wall). Real-time observations revealed lateral PspA and PspG complexes are highly mobile, but absent in cells lacking MreB. Without the MreB cytoskeleton, induction of the Psp response is still observed, yet these cells fail to maintain pmf under stress conditions. The two spatial subspecies therefore appear to be dynamically and functionally distinct with the polar clusters being associated with sensory function and the mobile complexes with maintenance of pmf.

## Introduction

The phage shock protein (Psp) response helps maintain the proton motive force (pmf) in cells under pmf-dissipating stress conditions (Kleerebezem *et al*., 1996; Jovanovic *et al*., 2006). The Psp response is found in many Gram-negative bacteria including several pathogens (Darwin, 2005) where Psp proteins are implicated in infectious processes. This presents a potential antibacterial drug target. For example, *pspC* mutants of *Yersinia enterocolitica* are severely attenuated for virulence during infection (Darwin and Miller, 1999) and *psp* genes are amongst the most highly upregulated genes in *Salmonella typhimurium* during macrophage infection (Eriksson *et al*., 2003). Psp is also upregulated during swarming in *S. typhimurium* (Wang *et al*., 2004) and during biofilm formation in *E. coli* (Beloin *et al*., 2004). The Psp response and PspA homologues have recently been found among Gram-positive bacteria, cyanobacteria, archea and higher plants (Darwin, 2005; Bidle *et al*., 2008; Vrancken *et al*., 2008) suggesting a fundamental role for Psp in maintaining cell envelope integrity throughout all three domains of life.

Transcription of the *psp* genes is driven via σ^54^-dependent promoters and requires activation by the enhancer binding protein PspF (Model *et al*., 1997; Darwin, 2005). The activity of PspF (and thus the PspF regulon) is regulated by PspA, which specifically interacts with PspF under non-inducing conditions (Model *et al*., 1997; Darwin, 2005). Upon induction, the interaction between PspA and PspF is disrupted, allowing activation of *psp* transcription (Lloyd *et al*., 2004; Darwin, 2005; Jovanovic *et al*., 2006). Psp is strongly induced by the outer membrane (OM) secretin protein IV (pIV) from filamentous phage f1 (Brissette *et al*., 1990), bacterial secretins and inner membrane (IM) stresses including conditions that block or reduce the efficiency of the protein export apparatus, extreme heat shock, hyper-osmotic shock and uncouplers of pmf (Model *et al*., 1997; Darwin, 2005).

PspA is a peripheral protein at the cytoplasmic face of the IM. It has been shown *in vitro* that PspA can directly interact with phosphatidylserine and phosphatidylglycerol (Kobayashi *et al*., 2007). PspG is predicted to be an integral IM protein (Lloyd *et al*., 2004) and is not known to have any major involvement in Psp transcription regulation. Instead, PspG, as well as PspA, are effectors of the Psp response (Jovanovic *et al*., 2006). Recently, work with *E. coli* IM vesicles and liposomes demonstrated that higher molecular-weight oligomers of PspA mitigate proton loss across membrane bilayers (Kobayashi *et al*., 2007). Transcription profiling of cells overexpressing PspA and PspG suggest these proteins cause a subtle switch towards anaerobic respiration and fermentation as well as a downturn of pmf consuming cellular processes such as motility (Jovanovic *et al*., 2006).

Although substantial progress has been made in understanding the regulation and function of the Psp response, its precise mechanisms as well as the spatial organization of Psp proteins within the living cell are still unknown. Although lacking the confined cellular compartments of eukaryotes, *in vivo* localization studies using fluorescence microscopy have revealed a remarkable organization within the bacterial cell. Based on asymmetric protein distribution, rod-shaped cells such as *E. coli* can be divided into different subcellular domains: the polar, lateral and septal regions. Correct spatial localization of proteins within these regions is often crucial for the functioning of cellular processes. Furthermore, members of interrelated pathways often colocalize, hence studying the spatial organization of a protein can provide insights into its role within the complex network of cell physiology. We have therefore visualized PspA and PspG in *E. coli* using a bright variant of the green fluorescent protein, GFPmut2 (herein termed GFP) (Cormack *et al*., 1996). Our subcellular studies allow us to probe the mode of action and the relationship between the Psp response and other cellular proteins and processes. Based on localizations of PspA and PspG and the determinants required for their correct localization, we identified different classes of Psp molecules associated with different roles in the Psp response and a link to cytoskeletal features of the cell. Additionally, we show, for the first time, that PspA forms higher-order oligomers *in vivo*.

## Results

### GFP–PspA and PspG–GFP are functional

GFP–PspA was created by fusing PspA to the C-terminus of GFP in pDSW209 (plasmid pEC1), and PspG–GFP was created by cloning *pspG* into pDSW210 (plasmid pGJ7) for an N-terminal fusion. To confirm their native state and to rule out artefacts due to accumulation of fluorescent aggregates or proteolytic cleavage products within inclusion bodies caused by overexpression (Ventura and Villaverde, 2006; Bardy and Maddock, 2007), we tested the stability, functionality and the extent of inclusion body formation.

**Fig. 1:**
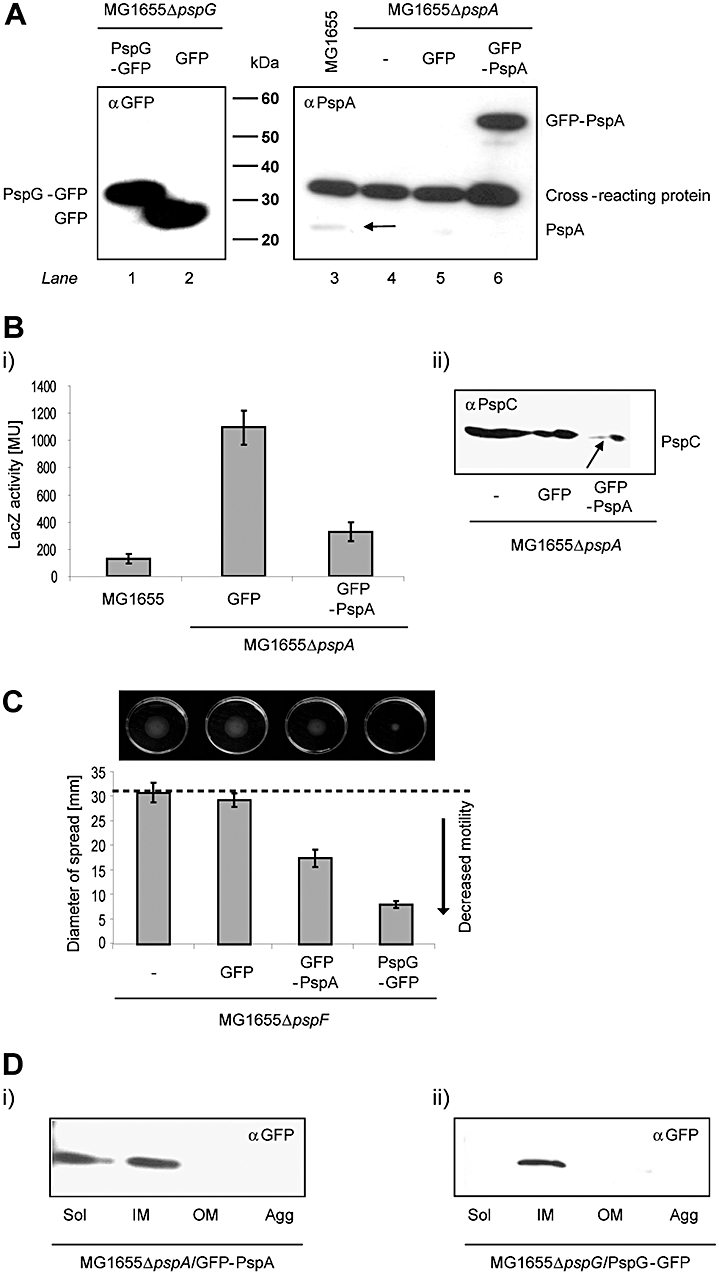
GFP–PspA and PspG–GFP are stable and functional. A. The stability of GFP–PspA and PspG–GFP fusion proteins were tested by immunoblotting with antibodies against PspA and GFP. Proteins corresponding to GFP–PspA (53 kDa, lane 6), PspG–GFP (37 kDa, lane 1), GFP (28 kDa) and PspA (25 kDa) are indicated. The PspA antibody cross-reacts with a 35 kDa *E. coli* protein (labelled cross-reacting protein). B. The negative regulatory function of GFP–PspA was (i) analysed by β-galactosidase assays in MG1655Δ*pspA* cells containing a *pspA-lacZ* transcriptional reporter fusion and (ii) by immunoblotting with PspC-specific antibodies, where GFP–PspA over-production causes a decrease in PspC levels (indicated by an arrow). C. The effector function of the GFP fusions were tested by motility assays using MG1655Δ*pspF* cells expressing either GFP–PspA or PspG–GFP (as described in *Experimental procedures*). D. A Triton X-100-based fractionation method was used to determine the location and amount of inclusion body formation of the fusion proteins. GFP–PspA (i) and PspG–GFP (ii) were detected by immunoblotting with GFP-specific antibodies. Sol (soluble fraction; cytoplasmic and periplasmic proteins), IM (inner membrane), OM (outer membrane) and Agg (aggregated) proteins (inclusion bodies).

Immunoblotting demonstrated that the expression level of GFP–PspA is very similar to that of the native PspA protein upon pIV-induced membrane stress (compare Fig. 1A and Fig. 1SA) and hence the fusions represent native Psp protein levels. Furthermore, the fusions appeared stably expressed. Neither MG1655Δ*pspA*/GFP–PspA nor MG1655Δ*pspG*/PspG–GFP cells contained detectable amounts of free PspA, PspG or GFP (Fig. 1A). PspA is a bifunctional protein (negative regulator and effector of Psp). The negative regulator function of GFP–PspA was tested using β-galactosidase assays in MG1655Δ*pspA*/GFP–PspA cells containing pSJ1 (φ*pspA-lacZ* transcriptional reporter fusion) (Fig. 1B) and immunoblotting with PspC-specific antibodies (Fig. 1B). GFP–PspA behaves very similarly to wild-type PspA when produced *in trans*, in partially inhibiting PspF activity and reducing PspC expression (compare Fig. 1B and Fig. S1B). The effector function of GFP–PspA was tested using a motility assay (Fig. 1C) because over-production of PspA in wild-type MG1655 or MG1655Δ*pspF* cells resulted in decreased motility (Jovanovic *et al*., 2006). Importantly, GFP–PspA in MG1655Δ*pspF* cells also caused decreased cell motility (Fig. 1C), demonstrating that GFP–PspA retains its effector functionality. Lloyd *et al*. (2004) identified PspG as an additional effector of the Psp response; over-production of PspG in wild-type MG1655 or MG1655Δ*pspF* cells also decreased cell motility. In line with this, expression of PspG–GFP in MG1655Δ*pspF* cells also resulted in decreased cell motility (Fig. 1C) demonstrating that the effector function of PspG–GFP is maintained.

Overexpressed GFP fusions can form fluorescent aggregates which reside in inclusion bodies potentially yielding artificial subcellular localization patterns (Bardy and Maddock, 2007). To test inclusion body formation of GFP–PspA and PspG–GFP we performed Triton X-100 based cell fractionations (Russel and Kazmierczak, 1993). Proteins from MG1655Δ*pspA*/GFP–PspA and MG1655Δ*pspG*/PspG–GFP cells were separated into soluble (periplasmic and cytoplasmic proteins) (Sol), IM, OM and aggregated fractions. Subsequently, the subcellular location of GFP–PspA and PspG–GFP was inferred via immunoblotting using GFP-specific antibodies (Fig. 1D). GFP–PspA can be detected in soluble and IM fractions (Fig. 1Di, Sol and IM), consistent with previous observations that PspA is a peripheral IM protein at the cytoplasmic face of the IM (Brissette *et al*., 1990). *In silico* predictions suggest PspG is an integral IM protein with two *trans*-membrane helixes and both the N- and C-terminus in the cytoplasm (Fig. S1). In agreement with this, PspG–GFP was only found in the IM fraction (Fig. 1Dii, IM), similarly to wild-type PspG (Fig. S1B) and neither GFP–PspA nor PspG–GFP showed any detectable aggregates (Fig. 1Di and ii, Agg, and Fig. S1B). We conclude that GFP–PspA and PspG–GFP are stably expressed, retain detectable levels of activity and are not located in inclusion bodies indicating that they are suitable for all localization studies.

### GFP–PspA and PspG–GFP are localized to the cell poles and traffic between both poles

**Fig. 2:**
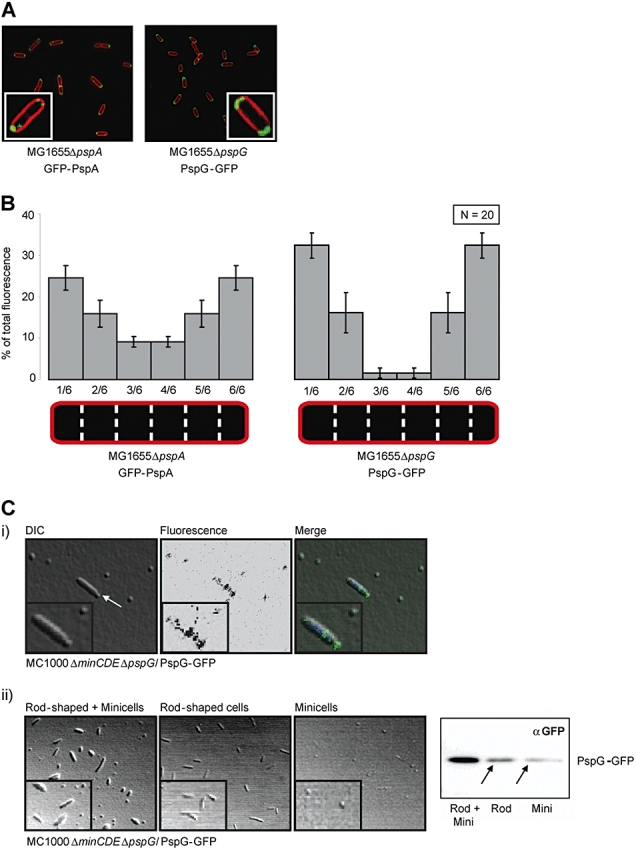
GFP–PspA and PspG–GFP localize at both cell poles. A. Epifluorescence images of *E. coli* MG1655Δ*pspA*/GFP–PspA and MG1655Δ*pspG*/PspG–GFP cells. Fluorescent GFP fusion proteins can be seen in green. The IM was stained with FM 5–95 (Molecular Probes; red). Images were taken using a Zeiss Axiovert 200 (inverted) microscope. B. The distribution of fluorescence intensity across six equally sized cross-segments (1/6–6/6) of 20 cells from each strain MG1655Δ*pspA*/GFP–PspA and MG1655Δ*pspG*/PspG–GFP was measured using ImageJ software. Values shown had background fluorescence substracted (measured using MG1655 cells treated with the FM 5–95 IM dye). C. *E. coli* MG1655Δ*minCDE*Δ*pspA*/GFP–PspA and MG1655Δ*minCDE*Δ*pspG*/PspG–GFP cells were (i) visualized using confocal fluorescence microscopy (the arrow indicates cell division at a misplaced division site close to one cell pole; DNA was stained using DAPI) and (ii) separated into rod-shaped and minicells (as confirmed by light microscopy), and using GFP-specific antibodies in the presence of the fusion proteins was assessed [arrows indicate PspG–GFP found in rod-shaped (Rod) and minicells (Mini)]. The results shown refer to MG1655Δ*minCDE*Δ*pspG*/PspG–GFP.

In line with their previously established topologies as peripheral (PspA) and integral IM proteins (PspG), GFP–PspA in MG1655Δ*pspA* and PspG–GFP in MG1655Δ*pspG* cells can be visualized as green fluorescent foci (Fig. 2A) localized proximal to the IM (Fig. 2A, red). Epifluorescence microscopy indicates that both, GFP–PspA and PspG–GFP (Fig. 2A, green) appear to accumulate at the cell poles. To quantify these localization patterns we divided 20 cells (of each strain used) into six equally sized cross-sections each covering similar membrane areas in the terminal and lateral sections (approximating the cell poles as hemispheres and assuming an average cell-length of 3 μm and a cell-diameter of 1 μm) and measured the intensity of the fluorescence signal in each segment (Fig. 2B). In accordance with the fluorescence images, in both MG1655Δ*pspA*/GFP–PspA and MG1655Δ*pspG*/PspG–GFP (Fig. 2A) cells almost half of the total fluorescence is accounted for by the polar segments 1/6 and 6/6 at the ends of the cells (Fig. 2B).

To explore the polar localization further we visualized GFP–PspA and PspG–GFP in *E. coli* MC1000Δ*minCDE* cells. The MinCDE system is required to maintain the placement of the divisome in mid-cell thereby resulting in two equally sized daughter cells after cell division (Lutkenhaus, 2007) (Fig. 2C.i). Cells lacking the MinCDE system are characterized by two different cell types, rod-shaped cells and minicells (Fig. 2C), due to the inappropriate placement of the division site close to one pole (Fig. 2Ci, differential interference contrast, arrow). Hence, minicells represent a polar segment of the parental cell harbouring polar localized proteins (Lai *et al*., 2004). Using confocal microscopy, we detected fluorescence in minicells using either MC1000Δ*minCDE*Δ*pspA*/GFP–PspA (data not shown) or MC1000Δ*minCDE*Δ*pspG/*PspG–GFP (Fig. 2Ci). As it is possible to separate minicells from rod-shaped cells by differential centrifugation (Lai *et al*., 2004) (Fig. 2Cii) we reasoned that detection of GFP–PspA and PspG–GFP in minicells by immunoblotting would further validate their polar localization. (Fig. 2Cii). Consistent with our earlier microscopy observation (Fig. 2A and Fig. 2Ci) that GFP–PspA and PspG–GFP are concentrated at the cell poles, both fusion proteins can be found in the minicells (Fig. 2Cii).

**Fig. 3:**
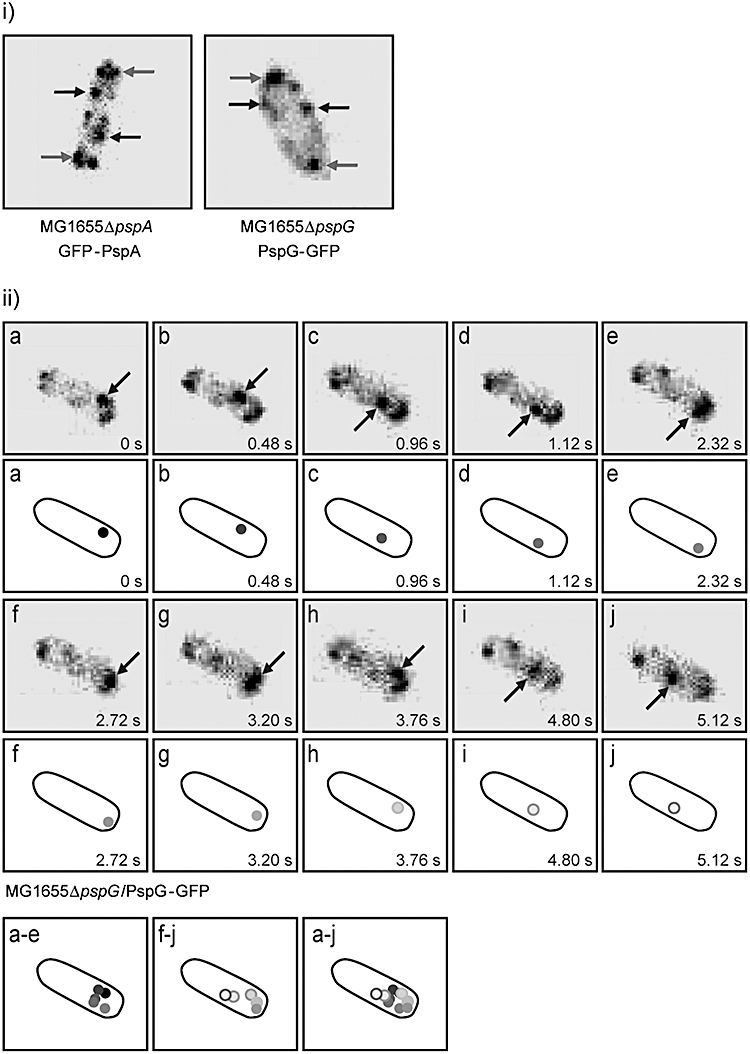
GFP–PspA and PspG–GFP traffic between the cell poles. (i) Inverted epifluorescence images of *E. coli* MG1655Δ*pspA*/GFP–PspA and MG1655Δ*pspG*/PspG–GFP cells. Polar GFP fusion proteins are indicated with grey arrows, lateral complexes with black arrows. (ii) Inverted time-lapsed epifluorescence images of MG1655Δ*pspG*/PspG–GFP cells. The movement of one of the mobile lateral complexes is indicated in each image by a black arrow. For clarification, schematic figures were drawn to illustrate the movement of the complex. The spot which represents the mobile fluorescent complex was shaded from black to light grey to indicate increasing time (i.e. black represents t = 0 s and white with grey outline represents t = 5.12 s). Composite figures were made to demonstrate the movement of the complex over time (a–e, f–j, a–j). All images were taken using a Nikon TE-2000 inverted optical microscope with a time resolution of 80 ms/frame. (For original images see *Supporting information* and the supplementary video clip.)

Although fluorescence intensity measurements across the cell indicated that a significant amount of fluorescence is located in the two segments at the ends of the cell (Fig. 2B, and 1/6 and 6/6), we still detected fluorescence between the poles (Fig. 2B, 2/6–5/6). Additionally GFP–PspA and PspG–GFP were also present in the rod-shaped MC1000Δ*minCDE* cells (Fig. 2C), implying that GFP–PspA and PspG–GFP may also be present laterally between the cell poles, just not easily visualized. Therefore, we examined MG1655Δ*pspA*/GFP–PspA and MG1655Δ*pspG*/PspG–GFP strains using an epifluorescence microscopy equipped with a tuneable argon ion laser in order to improve the resolution of the localization data. In addition to the polar foci (Fig. 3i, grey arrows) the images obtained clearly revealed lateral fluorescent complexes (Fig. 3i, black arrows) of both GFP–PspA and PspG–GFP. The non-uniform localization pattern along the lateral cell wall implies an underlying organization. Intriguingly, in marked contrast to the polar foci, the lateral complexes were highly mobile displaying a range of curved and linear motions (Fig. 3i, ii a–j, black arrows; supplementary data and supplementary video clip). These motions might represent the trafficking of PspA and PspG via an IM route, potentially dependent on the actin-like cytoskeletal protein MreB (Jones *et al*., 2001; Daniel and Errington, 2003; Shih *et al*., 2003; Figge *et al*., 2004; Gitai *et al*., 2004).

### GFP–PspA and PspG–GFP trafficking is affected by A22

To investigate a potential interaction of PspA and PspG with the MreB cytoskeleton, we studied the subcellular localization of GFP–PspA and PspG–GFP in the presence of A22 (S-(3.4-dichlorobenzyl)-isothiourea), a compound that induces MreB depletion-like spherical cell shape (Iwai *et al*., 2002). Gitai *et al*. (2005) and Kruse *et al*. (2006) showed that A22 treatment prevents MreB from forming a helical structure, which manifests in a delocalized fluorescence pattern of GFP–MreB. Remarkably, and in line with our proposal that PspA and PspG lateral complex formation and their trafficking may somehow involve the MreB cytoskeleton, we observed an A22-dependent change in the subcellular localization of GFP–PspA and PspG–GFP.

**Fig. 4:**
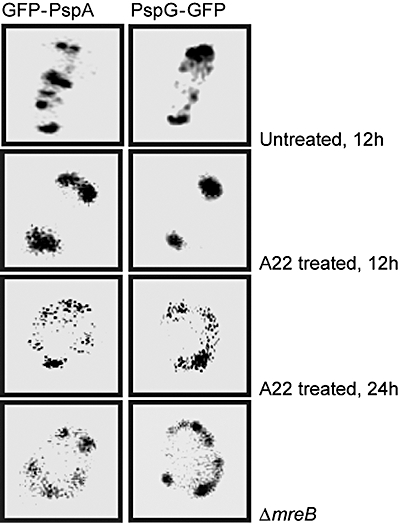
Lateral complexes of GFP–PspA and PspG–GFP are affected by A22 treatment. Inverted epifluorescence images of *E. coli* MG1655Δ*pspA*/GFP–PspA and MG1655Δ*pspG*/PspG–GFP cells after 12 h growth without A22 (untreated, 12 h), after 12 h (A22 treated, 12 h) and 24 h (A22 treated, 24 h) incubation with 50 μg ml^−1^ A22 demonstrate that the lateral complexes are affected by A22 treatment. Consistent with this, *E. coli* MC1000Δ*mreB* cells expressing either GFP–PspA or PspG–GFP also demonstrate that either disruption or removal of MreB results in only static polar GFP–PspA and PspG–GFP complexes being visible, the mobile lateral complexes (seen in Fig. 3) can no longer be observed. All images were taken using Nikon TE-2000 inverted optical microscope with a time resolution of 80 ms/frame. For each condition and strain, 60 cells were analysed. Each cell shown represents the localization pattern typical of > 90% of the population (for original images see *Supporting information*).

Recall that in untreated *E. coli* MG1655Δ*pspA* and MG1655Δ*pspG* cells, GFP–PspA and PspG–GFP are observed largely as polar foci (Fig. 2A), alongside highly mobile complexes between the poles (Fig. 3). After 12 h incubation with 50 μg ml^−1^ A22, the cells lose all detectable mobile lateral complexes but the foci on both poles remain unaffected (Fig. 4, A22 treated, 12 h). This loss of pole to pole trafficking is specific to A22 treatment and cannot be attributed to overnight cell growth as the localization pattern in these cells is unchanged and similar to untreated cells (Fig. 4, untreated, 12 h). In agreement with A22 affecting the MreB polymerization (Iwai *et al*., 2002; Gitai *et al*., 2005), we observed formation of spherical cells after 24 h growth in the presence of A22, confirming that the MreB helical structure is disrupted (Fig. 4, A22 treated, 24 h). Our findings indicate that both PspA and PspG not only show two spatially (polar and lateral) distinct localizations, but also the mechanisms behind the positioning of the polar and lateral complexes may differ. While the polar complexes appear to be A22 resistant and therefore MreB independent, the mobile lateral spots delocalize upon A22 treatment. Hence, PspA and PspG might rely directly upon the MreB cytoskeleton to properly form lateral complexes and move between the cell poles, and the organization of the MreB cytoskeleton could explain the predominantly curved profile of that route (Fig. 3). Imaging of GFP–PspA and PspG–GFP in cells lacking *mreB* (MC1000Δ*mreB*) supported our observations using A22, in which lateral movements were no longer observed and the fluorescent foci appeared dispersed (Fig. 4, Δ*mreB*). A similar pattern was seen by Shih *et al*. (2005) with polar Tar-GFP in MC1000Δ*mreB,* which the authors concluded may imply that polar localization is dependent on MreB. However, we could not distinguish whether the delocalized GFP–PspA and PspG–GFP in MC1000Δ*mreB* (Fig. 4) corresponds to either the polar or lateral complexes observed in wild-type *E. coli* cells (Fig. 3i). The resistance of polar foci to A22, however, suggests MreB-independent positioning of PspA and PspG at the cell pole.

**Fig. 5:**
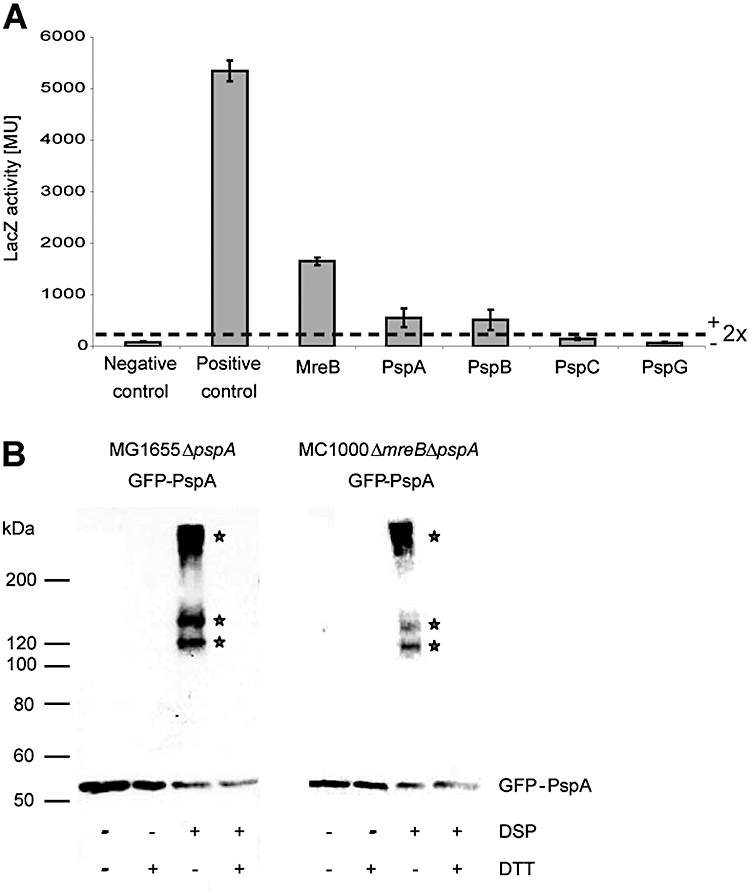
Weak or transient interactions occur between PspA or PspB and MreB. A. Pair-wise interactions of MreB with MreB, PspA, PspB, PspC or PspG were tested using the *cya*-based bacterial two-hybrid (BACTH) assay (Karimova *et al*., 1998). A more than twofold increase in the LacZ activity above the negative control level (T18 + T25) (indicated as dashed line) was scored as positive interaction. Positive control: T18zip + T25zip. B. *In vivo* protein cross-linking in GFP–PspA expressing MG1655Δ*pspA* and MC1000Δ*mreB* cells indicates the presence of three new cross-linked species (asterisks) which were determined to contain GFP–PspA using GFP-specific antibodies. The specificity of the cross-linked species was confirmed using the reducing agent DTT.

To determine whether a direct interaction between the Psp system and MreB is occurring, we performed *cya*-based bacterial two-hybrid assays (BACTH) (Karimova *et al*., 1998) with PspA, PspB, PspC and PspG (Fig. 5A). Pair-wise interactions of the two proteins of interest were assessed by measuring the β-galactosidase (LacZ) activity. An interaction was scored as positive if the LacZ activity was twofold higher (approximately 252 MU) than the negative control, pUT18C + pKT25 (84 ± 13 MU). MreB is known to interact with itself, thereby forming a helical polymer (Jones *et al*., 2001), which was used as a positive control to score for the functionality of the MreB fusion proteins (T18-MreB and T25-MreB) and was measured as a significant increase in the LacZ activity (1653 ± 75 MU). Consistent with the subcellular delocalization of PspA, in the presence of A22 and in MC1000Δ*mreB* cells, the average LacZ activity of cells expressing T18-PspA and T25-MreB (559 ± 183 MU) was well above the twofold threshold over the negative control value. Co-expression of T18-MreB and T25-PspB also significantly increased the LacZ activity (521 ± 195 MU). Hence, the data indicate that PspA and PspB associate with MreB, potentially interacting directly or as part of a complex containing additional factors. However, PspC (147 ± 17 MU) and PspG (75 ± 13 MU) do not appear to directly interact with MreB. PspG has been observed to co-purify with PspA (L.J. Lloyd, unpubl. data), suggesting that PspG might indirectly interact with MreB via PspA, potentially explaining the loss of lateral trafficking of PspG upon A22 treatment. To exclude the possibility of false negatives, the BACTH interactions of PspA with PspB and PspC were also tested (data not shown). The results obtained were consistent with previous findings where PspA interacts with PspB and PspC (Adams *et al*., 2003), confirming that the lack of interactions with MreB were not the result of misfolded protein fusions.

To further explore the potential interaction between PspA and MreB (suggested by the BACTH assay) we compared the *in vivo* cross-linking pattern of GFP–PspA in *E. coli* MG1655Δ*pspA*/GFP–PspA and MC1000Δ*pspA*Δ*mreB*/GFP–PspA cells, using the thiol-reactive agent dithiobis(succinimydylpropionate), DSP (PIERCE) (as described in *Experimental procedures*). Following SDS-PAGE, the cross-linked GFP–PspA protein complexes were visualized using antibodies against GFP (Fig. 5B). In addition to monomeric GFP–PspA (53 kDa), in MG1655Δ*pspA*/GFP–PspA and MC1000Δ*mreB*/GFP–PspA cells treated with DSP we observed three higher-molecular-weight bands (Fig. 5B, indicated by asterisks). These bands could no longer be detected in the presence of DTT (which specifically cleaves the cross-linker) and were absent in the negative controls, MG1655Δ*pspA*/GFP and MC1000Δ*mreB*/GFP (data not shown), indicating that these species are indeed cross-linked GFP–PspA complexes. We reasoned that if a direct interaction between MreB and PspA was occurring, then one of these cross-linked species (Fig. 5B) could represent GFP–PspA–MreB. In cells lacking MreB (MC1000Δ*mreB*Δ*pspA*/GFP–PspA) we observed a quantitative change to the cross-linking pattern in the presence of DSP compared with cells harbouring the chromosomal *mreB* gene (MG1655Δ*pspA*/GFP–PspA), with the middle cross-linked species present at a consistently lower level (Fig. 5B). These data indicate that MreB (either directly or indirectly) contributes to the formation of complexes containing GFP–PspA, although a single stable MreB–PspA complex is not resolved.

### Psp induction and maintenance of pmf are spatially separated

#### 

##### Polar localized Psp proteins are sufficient to support Psp induction

**Fig. 6:**
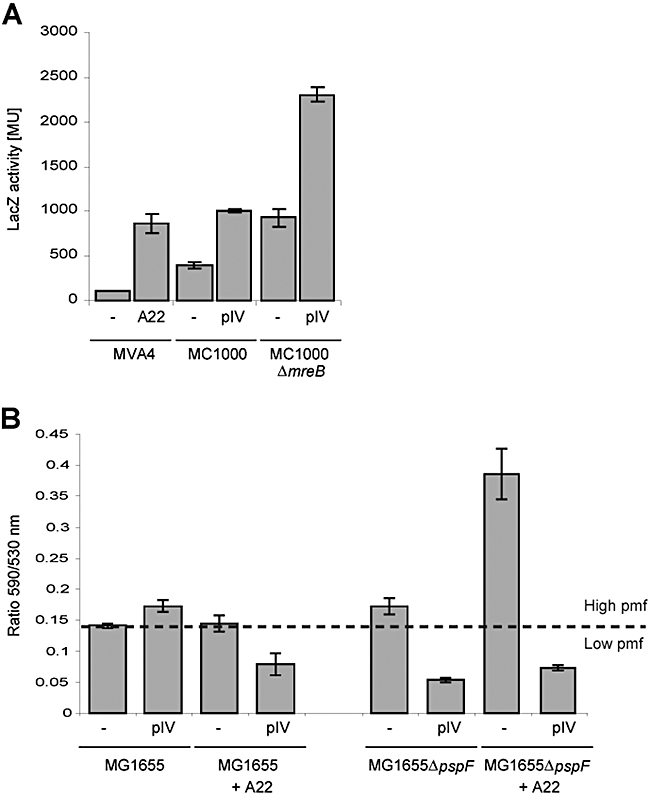
Psp induction and maintenance of pmf are spatially separated. The effect of impaired trafficking of Psp proteins was analysed in *E. coli* cells treated with A22 and deleted for *mreB*. A. Induction of the Psp response was measured using β-galactosidase assays and a chromosomal Φ*pspA-lacZ* transcriptional reporter fusion. Both A22 treatment and deletion of *mreB* significantly induce Psp transcription. B. Maintenance of pmf in cells lacking helical Psp trafficking was tested using the electron potential indicator dye JC1 (Jovanovic *et al*., 2006). Pmf was measured as the ratio between red and green cells averaged across three different microscopic fields. Increase in ratio values corresponds to an increase in electron potential.

It has been shown recently that A22 inhibits peptidoglycan synthesis during elongation (Uehara and Park, 2008). This might be the consequence of impaired MreB polymerization and may by itself cause IM stress to the cell. In line with this, we found that both, A22 and *mreB* gene deletion induce the Psp IM stress response as measured by the β-galactosidase activity from cells harbouring a chromosomal *pspA* promoter *lacZ* fusion (Fig. 6A). Importantly a further increase in *pspA-lacZ* expression was observed following induction of pIV in the Δ*mreB* mutant. Together with our epifluorescence images where GFP–PspA is found only at the poles upon A22 treatment (Fig. 4), we suggest that signalling, sensing and the release of the PspA–PspF inhibitory complex could occur at the cell poles if a cytoplasmic and regulatable PspA–PspF complex does not exist.

##### MreB-dependent trafficking of PspA and PspG is important for pmf maintenance under pIV-secretin stress

Since the Psp system is induced by stresses which interfere with cell envelope integrity and MreB is involved in peptidoglycan synthesis (Osborn and Rothfield, 2007), we considered that the potential trafficking of PspA and PspG along the MreB cytoskeleton coincides with a direct role of the Psp response in cell wall biosynthesis, which in turn might help maintain the pmf of stressed cells. We probed the relationship between the effector functions of PspA and PspG and the lateral complexes by measuring pmf conservation under pIV-secretin stress in cells treated with A22 (Fig. 6B) and in cells deleted for the MreB cytoskeleton (MC1000Δ*mreB*) (Supplementary Fig. 5).

Wild-type *E. coli* cells demonstrate no change in pmf upon pIV-induced IM stress, presumably due to the action of the Psp effectors. Stressed cells lacking the transcriptional activator PspF are unable to produce Psp proteins and therefore fail to maintain wild-type pmf levels. The pmf in wild-type *E. coli* cells treated with A22 or cells deleted for *mreB* remains high. In contrast to pIV secretin, A22 does not cause a drop in pmf in cells deleted for PspF (MG1655Δ*pspF* A22) but rather causes an increase in pmf. This suggests that some targets of A22, including the Psp effector proteins, will function in pmf homeostasis. When Psp proteins are induced by pIV, in cells treated with A22 or deleted for *mreB* (each a condition where lateral Psp proteins will be lacking) pmf is significantly decreased. This decrease in pmf is strikingly similar to MG1655Δ*pspF* cells expressing pIV, which are unable to mount a Psp response. The pmf drop in pIV expressing cells lacking the lateral Psp complexes implies that the loss of helical Psp trafficking is important for maintaining pmf under pIV-induced stress (Fig. 6B). Interestingly, cells deleted for *pspF* show a significant increase in pmf upon A22 treatment compared with wild-type cells, which may further relate to the absence of lateral Psp protein complexes.

## PspA forms two oligomeric subclasses *in vivo*

**Fig. 7:**
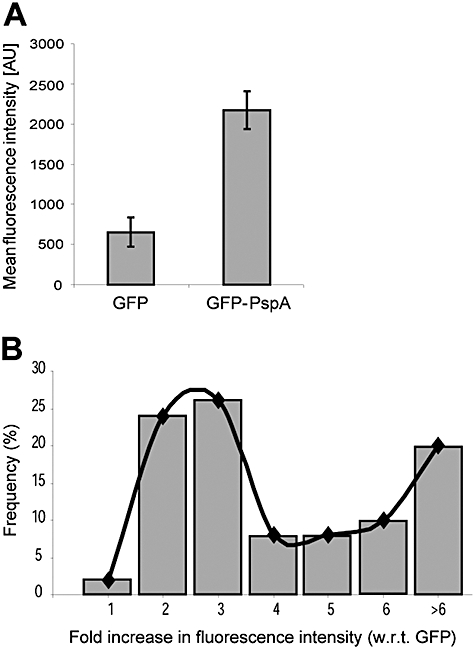
GFP–PspA forms high-order oligomers *in vivo*. Analysis of the *in vivo* oligomerization state of PspA was performed by single molecule fluorescence imaging using objective-type total internal reflection fluorescence (TIRF) on a Nikon TE-2000 inverted optical microscope. The number of molecules in fluorescent complexes was estimated using the ImageJ software. The intensity of a single pixel in a fluorescent GFP–PspA cluster expressed in *E. coli* MG1655Δ*pspA* cells was measured and compared with GFP from cell lysates of *E. coli* MG1655 cells harbouring pDSW209. A. The mean fluorescence intensity of 50 GFP–PspA complexes within living cells was on average at least three times higher than GFP spots, suggesting that PspA can form higher oligomers *in vivo*. B. The frequency distribution among the 50 complexes analysed showed that GFP–PspA self-assembles into two distinct oligomeric subclasses.

According to gel-filtration chromatography profiles (Kobayashi *et al*., 2007) and structural studies (Hankamer *et al*., 2004), PspA can form high-order oligomers *in vitro*. Kobayashi *et al*. (2007) also showed that oligomeric PspA can directly interact with lipids and block proton leakage of membrane vesicles and liposomes. We estimated the number of molecules in a fluorescent cluster of GFP–PspA by measuring its relative fluorescence intensity within living cells, using single molecule fluorescence imaging and objective-type total internal reflection fluorescence (TIRF), and compared this with the fluorescence intensity of GFP foci from cell lysates of *E. coli* MG1655 harbouring pDSW209. We show for the first time that PspA forms oligomers *in vivo* (Fig. 7), supporting the previous *in vitro* findings on the structure of PspA complexes. We found two distinct oligomeric subclasses of PspA within the living cell. Approximately half of the 50 complexes analysed appeared to have two- to three-fold higher fluorescence intensities than GFP alone (Fig. S2B). The second subclass (∼20%) exhibited ‘off-scale’ fluorescence intensities, which were clearly more than sixfold above GFP alone. We note however that the TIRF mode may underestimate the number of proteins in the oligomer, because (i) the imaged complex may not be optimally positioned to record its full fluorescence (the TIRF field decays exponentially with distance from the interface and the membrane surface may be a significant distance away from the microscope slide) and (ii) some complexes were larger than those quantitatively analysed, referred to as ‘off-scale’. It is evident however that PspA self-assembles in different oligomeric states *in vivo*.

## Discussion

Studying the spatial organization of a protein can provide insights into its function and integration within the network of cellular processes. We have visualized, for the first time, GFP-labelled PspA and PspG *in vivo*, two members of the Psp response, in *E. coli* using a combination of epi-, confocal and TIRF microscopy. Functional assays with these fusion proteins indicate that the information obtained in this work faithfully represents the native organization of PspA and PspG within the living *E. coli* cell. This study permitted the analysis of the mode of action and the relationship between the Psp response and other proteins and cellular processes and revealed distinct classes of Psp molecules and a link to cytoskeletal features of the cell.

### GFP–PspA and PspG–GFP accumulate at the cell poles

Using fluorescence microscopy, PspA and PspG fusion proteins were observed to localize at cell poles. Polar localization was confirmed using cells deleted for the MinCDE system (*E. coli* MC1000Δ*minCDE*), in which we found both GFP–PspA and PspG–GFP within minicells (which resemble the polar region of the cell). The accumulation of PspA and PspG at the poles was unaffected by A22, suggesting that polar positioning is independent of the MreB cytoskeleton. As discussed below, being localized at the cell pole could be important for the function of PspA and PspG.

Previously we observed that deletion of either PspA or PspG significantly increased the expression of proteins involved in chemotaxis and motility, e.g. the methyl-accepting chemotaxis proteins Tar, Tap and Tsr (Jovanovic *et al*., 2006). These chemoreceptors are part of super-molecular complexes located in the IM at the cell pole (Maddock and Shapiro, 1993) where they sense environmental cues such as attractants or repellents and co-ordinate the chemotactical behaviour of the cell by adjusting the response of the pmf-consuming flagellar motor. Polar localized Psp proteins might act in concert with other polar proteins such as chemoreceptors to govern sensory inputs for pmf-consuming key cell properties such as motility, potentially in response to the energy state of the cell.

The Psp system is also implicated in virulence of pathogenic enterobacteria (Darwin, 2005). For example, Psp mutants of *Y. enterocolitica* show growth defects and attenuated virulence upon mislocalization of the secretin from the type III secretion system (Darwin and Miller, 2001), and macrophage infection highly induces *psp* transcription in *Salmonella enterica* and *Shigella flexneri* (Eriksson *et al*., 2003; Darwin, 2005). Remarkably, two proteins involved in *Shigella* infection, IcsA (enabling intracellular movement within the mammalian host) and IpaC (a component of the type III secretion system) are also found at the pole (Goldberg *et al*., 1993; Ebersbach and Jacobs-Wagner, 2007; Jaumouille *et al*., 2008). It is therefore conceivable that accumulation of Psp proteins at the cell pole is linked to their proposed role in promoting enterobacterial infections.

Interestingly, Strauss *et al*. (2008) proposed that local differences exist in the pH gradient across the IM cristae in mitochondria. According to their model, although a constant electrochemical potential exists along the entire IM surface, the local pH gradient, and hence the ΔpH contribution to the pmf, is significantly higher in regions of high-membrane curvature (Strauss *et al*., 2008). Applied to bacteria, this model suggests that both poles in rod-shaped cells might serve as proton sinks due to the increased pH gradient in this area. Protons leaking into the cytoplasm as a result of impaired IM integrity due to envelope stress might therefore preferentially accumulate at the cell poles. As the contribution of ΔpH to the pmf appears to be higher in this region, even small changes in the ΔpH might have a stronger effect on pmf at the poles than lateral regions of the cell. Hence, the poles might be the most sensitive part of the cell to measure changes in the electrochemical gradient. Polar localization of Psp proteins could therefore be important to perceive subtle changes in pmf and subsequently allow for a quick response to IM stress. Importantly, when we use A22 to disrupt the lateral complexes (Fig. 4) we demonstrate that the polar complexes alone are sufficient to mount a ‘Psp response’ in terms of signalling, sensing and release of PspA from PspF (Fig. 6A).

### MreB-dependent lateral complexes and the trafficking of GFP–PspA and PspG–GFP

In addition to the polar localization of GFP–PspA and PspG–GFP, fluorescence intensity measurement across the cell and their detection in rod-shaped *E. coli* MC1000Δ*minCDE* cells indicated the presence of PspA and PspG along the length of the cell. Due to the movements they displayed, we reasoned that the MreB cytoskeleton might be involved in the lateral organization of PspA and PspG. Using A22 and an *E. coli* strain lacking MreB, we demonstrated that lateral GFP–PspA and PspG–GFP complexes were no longer visible, implying MreB assists lateral Psp complexes, possibly through their formation, maintenance and potentially movements. The latter is reflected in the path taken (Fig. 3) which resembles the MreB cytoskeletal structure. Although BACTH and *in vivo* cross-linking analyses could not establish strong stable direct interactions between Psp proteins and MreB, our data do clearly indicate that PspA as well as PspB might interact weakly/transiently or indirectly with MreB. We propose that the mobility of the lateral complexes of GFP–PspA and PspG–GFP could be one consequence of these weak/transient interactions with the MreB cytoskeleton, which could serve as a scaffold supporting rapid trafficking across the entire cell. As MreB is thought to have a role in cell wall biogenesis (Kruse *et al*., 2005) and the trafficking of Psp proteins appears MreB-dependent, we suggest that Psp effectors may also have a role in cell envelope biosynthesis. This view is supported by findings showing that Psp is induced upon inhibition of lipid biosynthesis in *E. coli*: (Bergler *et al*., 1994) and by cell wall biosynthesis defects in a *glmS* mutant of *Y. enterocolitica* (Maxson and Darwin, 2004).

High-order oligomers of PspA have been proposed to evenly coat the cytoplasmic face of the IM to restore membrane integrity (Kobayashi *et al*., 2007). Our subcellular localization studies of PspA, however, do not entirely support all aspects of this model. Although our results establish for the first time that PspA forms oligomers *in vivo*[broadly in line with previously reported *in vitro* data (Hankamer *et al*., 2004; Kobayashi *et al*., 2007; Standar *et al*., 2008)], PspA does not evenly coat the IM of the live cell. Instead of uniformly covering the entire cell, PspA (and PspG) is highly organized into what appear to be distinct functional classes (polar and lateral complexes). Together they may maintain the pmf of the cell by regulating pmf consuming processes such as motility and cell envelope biogenesis, as well as by directly restoring membrane integrity. The PspA and PspG complexes display a dynamic character when in association with the IM, and presumably make interactions with cellular components, in addition to lipids, extending activities beyond their role of binding lipids to ‘plug’ holes within the IM.

## Experimental procedures

### Bacterial strains, media and growth conditions

Bacterial strains and plasmids are listed in Table S1. Unless otherwise stated, strains were grown in Luria–Bertani (LB) broth or on plates at 37°C (Miller, 1992). For fluorescence microscopy, strains harbouring GFP fusions were grown in minimal medium [50 mM MOPS (pH 7), 2 mM MgSO_4_, 0.5% Glucose (w/v), 10 mM NH_4_Cl, 0.75 mM Na_2_SO_4_, 1.2 mM NH_4_NO_3_, 0.5 mM KH_2_PO_4_] at 30°C to minimize background fluorescence and allow correct folding of the GFP protein. Growth media for MC1000 strains were supplemented with 25 μg ml^−1^ leucine and 5 μg ml^−1^ thiamine*.* Where specified, protein expression was induced with 0.1 or 1.0 mM IPTG.

### DNA manipulations

Transductions were carried out by P1_*vir*_ phage as described by Miller (1992). *pspA* was amplified from the *E. coli* MG1655 chromosome using primer set GFP–PspA (Table S2) and cloned into pDSW209 (pLL5). GFP–PspA expressed from pLL5 was not functional, with respect to PspA activity, and so to recover functionality a serine/glycine linker sequence was introduced between GFP and PspA using site-directed mutagenesis with primer set Mut1 (Table S2) to create pEC1. *pspG* was amplified from *E. coli* MG1655 chromosomal DNA using primer set GFP–PspG (Table S2) and cloned into pDSW210. GFP–fusion proteins were produced by leaky expression under the control of a weak P_*trc*_ promoter, downregulated by the constitutive LacI^q^ repressor encoded on the plasmid (Weiss *et al*., 1999). For BACTH analysis, *mreB* was amplified from the *E. coli* MG1655 chromosome via primer set MreB1 or MreB2, and cloned into pUT18C and pKT25. Primer sequences are shown in 5′→ 3′ (Table S2). All constructs were verified by sequencing and protein expression was tested by immunoblotting using corresponding antibodies.

### Cell fractionation using Triton X-100

GFP fusion proteins were expressed in minimal media at 30°C and fractionated into soluble (cytoplasmic and periplasmic), IM, OM and insoluble components (Agg) as described (Brissette *et al*., 1990; Russel and Kazmierczak, 1993). The GFP fusion proteins were detected by immunoblotting with antibodies against GFP, PspA and PspG. The cell fractionation method was validated using SecA and TatA as marker proteins (Fig. S2).

### Immunoblotting

Immunoblotting was performed according to Elderkin *et al*. (2002) using αGFP antibodies (Clontech, BD) 1:8000 and αmouse secondary antibody 1:8000, αPspA (Jones *et al*., 2003) (1:10 000 and αmouse secondary antibody 1:10 000), αPspC (a gift from P. Model) (1:1000 and αrabbit secondary antibody 1:5000) or αPspG (Jovanovic *et al*., 2006) (1:1000 and αrabbit secondary antibody 1:5000).

### β-Galactosidase assay

Cells were grown overnight at 30°C in minimal medium containing the appropriate antibiotic and diluted 100-fold into the same medium. At OD_600_∼0.4 LacZ activity of the cells was measured as described (Miller, 1992).

### Motility assay

Motility assays were performed as described (Lloyd *et al*., 2004). Briefly, 2 μl of an overnight LB culture was pipetted onto soft agar plates [1% tryptone (w/v), 0.5% NaCl (w/v) and 0.3% agar (w/v)] containing the appropriate antibiotic and 1 mM IPTG where required. Plates were incubated overnight at room temperature, and zones of motility were measured in mm. Each strain was assayed six times. Standard errors of the mean are indicated.

### Fluorescence microscopy

A mid-log phase (OD_600_∼0.5) culture grown in minimal medium supplemented with the appropriate antibiotics was mounted onto a microscope slide prepared as described (Jovanovic *et al*., 2006). Where indicated, cell membranes were stained by adding 1 μl FM 5–95 dye (Molecular Probes) to 9 μl cells and DNA was stained using DAPI (300 nM) (Molecular Probes). Confocal fluorescence images were taken using a Leica SP2 upright microscope fitted with a HCX PL APO CS 63.0 × 1.32 Oil Ph3 objective. Epifluorescence images were taken using inverted epifluorescence microscopes and either a Zeiss Axiovert 200M fitted with a Plan-Neofluar objective (Zeiss 100x/1.30 Oil Ph3), a 300W xenon arc-lamp transmitted through a liquid light guide (Sutter Instruments), a Sony CoolSnap HQ cooled CCD camera (Roper Scientific), modified magnetron ET filter sets (Chroma); or a Nikon TE-2000 microscope equipped with a CoolView EM 1000/TV camera (Photonic Science) and a tuneable argon ion laser. Digital images were acquired and analysed using either METAMORPH (version V.6.2r6) or ImageJ software (http://rsbweb.nih.gov/ij/).

### Fluorescence microscopy of A22-treated cells

*Escherichia coli* MG1655Δ*pspA*/GFP–PspA and *E. coli* MG1655Δ*pspG*/PspG–GFP cells were grown in minimal medium at 30°C until OD_600_∼0.3. After 12 or 24 h incubation with 50 μg ml^−1^ of A22 (suspended in 100% methanol), the cells were immobilized with 0.1% (w/v) poly-l-lysine and spotted onto a cover-glass. PspA–GFP and PspG–GFP were visualized using epifluorescence microscopy. 0.1% (w/v) poly-l-lysine had no significant effect on the Psp response as judged by β-galactosidase assays (data not shown).

### Separation of mini and rod-shaped cells

*Escherichia coli* YLS1 (MC1000Δ*minCDE*) (a gift from Y-L. Shih) cells harbouring either GFP–PspA or PspG–GFP were grown in minimal medium supplemented with 25 μg ml^−1^ leucine and 5 μg ml^−1^ thiamine at 30°C. At mid-log phase, the cells were separated as described (Lai *et al*., 2004) and proteins visualized by immunoblotting with αGFP antibodies.

### Bacterial two-hybrid analysis

The *cya*-based BACTH system was performed as described (Karimova *et al*., 1998). BTH101 cells were co-transformed with plasmids encoding the protein of interest fused to either the T18 or T25 fragments of adenylate-cyclase (Cya) from *Bordetella pertussis*. Cells were grown at 30°C and expression of the fusion proteins induced by 0.5 mM IPTG. After 1 h the LacZ activity was measured by β-galactosidase assays, performed in triplicate per strain tested. A positive interaction was scored as a ≥ 2-fold increase in LacZ activity compared with the negative control (BTH101 cells with the T18 and T25 vectors alone). As a positive control we assayed cells containing pUT18C-zip and pKT25-zip plasmids carrying a fused GCN4 leucine-zipper sequence (Karimova *et al*., 1998).

### *In vivo* cross-linking

*In vivo* cross-linking was performed as described (Adams *et al*., 2003) using the thiol-reactive cross-linking agent dithiobis(succinimydylpropionate) DSP (PIERCE). Cells were grown in minimal medium at 30°C to an OD_600_ of 1.0, harvested and washed in 0.9% NaCl. Proteins were exposed to the cross-linker for 30 min (100 μM DSP in 125 mM HEPES (pH 7.3) at 25°C). To stop the reaction, 50 μl of 1 M Tris-HCl (pH 8.0) were added and the reaction was transferred to ice for 5 min. Proteins were separated on a 7.5% SDS-gel (run at 200 V for 50 min) and cross-linked species detected using αGFP antibodies.

### Proton motive force measurement

Proton motive force measurements were performed as described (Jovanovic *et al*., 2006) using the dye JC-1 (Molecular probes). The pmf was calculated as a ratio of red and green cells averaged across three different microscopic fields.

### Single molecule fluorescence imaging of PspA *in vivo*

Single molecules in fluorescent PspA complexes *in vivo* were imaged using objective-type TIRF on a Nikon TE-2000 inverted optical microscope equipped with a CoolView EM 1000/TV camera (Photonic Science) and a tuneable argon ion laser. *E. coli* MG1655 cells harbouring pDSW209 (GFP) and *E. coli* MG1655Δ*pspA*/GFP–PspA (for GFP–PspA) cells were grown in minimal medium at 30°C until OD_600_∼0.6. GFP cell lysates were prepared by sonication [50 mM Tris-HCl (pH 8.0), 100 mM NaCl, 1 mM EDTA (pH 8.0), 1 mM DTT]. GFP cell lysates and GFP–PspA expressing cells were mounted on cover glass and images were taken at 80 ms per frame and analysed manually using the ImageJ software.
