# Supplementary material for: In vivo localizations of membrane stress controllers PspA and PspG in Escherichia coli
Source: Mol Microbiol. 2009 Jun 30;73(3):382–96. doi: 10.1111/j.1365-2958.2009.06776.x (PMC2763126; doi:10.1111/j.1365-2958.2009.06776.x)
Supplement: Supplementary file 1 [file mmi0073-0382-SD1.pdf]

Supplementary information:

Tables

Table 1: *E. coli* K-12 strains and plasmids used in this study.

Strain or plasmid	Relevant characteristics	Reference
**Strain**		
MC1061	hsdR2 hsdM+ hsdS+ araD139 Δ(ara-leu)7697 Δ(lac)X74 galE15 galK16 rpsL (StrR) mcrA mcrB1	CGSC# 6649
MG1655	F^- λ^- ilvG- rfb-50 rph-1	CGSC# 7740
MG1655D*pspA*	Δ*pspA*	Lloyd *et al.*, 2004
MVA4	MC1061 Φ(pspA-LacZ) (ap^r)	This work
MVA40	MG1655Δ*pspG*	Lloyd *et al.*, 2004
YLS1	MC1000Δ*minCDE*	A gift from Yu-Ling Shih
YLS2	MC1000Δ*mreB*	A gift from Yu-Ling Shih
**Plasmids**		
pGZ119EH	IPTG-inducible *tac* promoter expression vector, (cam^r)	A gift from M. Russel
pPMR129	pGZ119EH harbouring *gIV* (pIV), (cam^r)	A gift from M. Russel
pDSW209	IPTG-inducible P_{trc} promoter *gfpmut2*-MCS fusion vector (ap^r)	Weiss *et al.*, 1999
pDSW210	IPTG-inducible P_{trc} promoter MCS-*gfpmut2* fusion vector (ap^r)	Weiss *et al.*, 1999
pLL5	pDSW209 harbouring *pspA* (ap^r)	This work
pEC1	pLL5 containing –GGGGSGGS- linker region between *pspA* and *gfp* (ap^r)	This work
pGJ7	pDSW210 harbouring *pspG* (ap^r)	This work
pMR25	*lacZ* transcriptional fusion vector (tet^r)	Jones *et al.*, 2003
pSJ1	pMR25 harbouring *pspA* promoter region (tet^r)	Jones *et al.*, 2003
pLL11	pBAD18c habouring *pspG* (kn^r)	Lloyd *et al.*, 2004
pUT18	pUC19 encoding T18 (aa 225-339 of CyaA) for N-terminal fusion (ap^r)	Karimova *et al.*, 1998

pUT18C	pUC19 encoding T18 (aa 225-339 of CyaA) for C-terminal fusion (ap^{r})	Karimova *et al.*, 1998
pUT18Czip	pUT18C encoding leucine zipper of GCN4 (ap^{r})	Karimova *et al.*, 1998
pKT25	pSU40 encoding T25 (aa 1-224 of CyaA) for C-terminal fusion (kn^{r})	Karimova *et al.*, 1998
pKNT25	pSU40 encoding T25 (aa 1-224 of CyaA) for N-terminal fusion (kn^{r})	Karimova *et al.*, 1998
pKT25zip	pKT25 encoding leucine zipper of GCN4 (kn^{r})	Karimova *et al.*, 1998
pAJM26	pKT25 harbouring *pspA* (kn^{r})	A gift from A. Mayhew
pAJM27	pKT25 harbouring *pspC* (kn^{r})	A gift from A. Mayhew
pAJM33	pUT18 harbouring *pspB* (ap^{r})	A gift from A. Mayhew
pEC2	pKT25 harbouring *mreB* (kn^{r})	This work
pEC3	pUT18C harbouring *mreB* (ap^{r})	This work
pEC4	pUT18 harbouring *pspG* (ap^{r})	This work

<u>Table 2: Primer sets used in this study.</u>

**Primer Set**	**Sequence (5' → 3')**
GFP-PspG	ccggaattccggatgctggaactactttttgtgattggc<br>cgcggatccgcggtaacgccagcggtcataacgc
GFP-PspA	cggaattcatgggtatttttctcgctt<br>cccaagcttttattgattgtcttgcttcat
Mut1	Gcatggatgaactatacaaaggtggtggtggttctggtggttctgaattcatgggtatttttctc<br>gagaaaaaatacccatgaattcagaaccaccagaaccaccaccacctttgtatagttcatccatgc
MreB1	agctaggtctagagatgttgaaaaaatttcgtgg<br>agctgttggatccgattactcttcgctgaacaggtcg
MreB2	agctaggtctagagatgttgaaaaaatttcgtgg<br>agctgttggatccgactcttcgctgaacaggtcg
PspG-T18	agctaggtctagagatgctggaactactttttgtgattggcttt<br>gctgttggtacccagtaacgccagcggtcataacgctgatattt

Supplementary Figures

Supplementary Figure 1

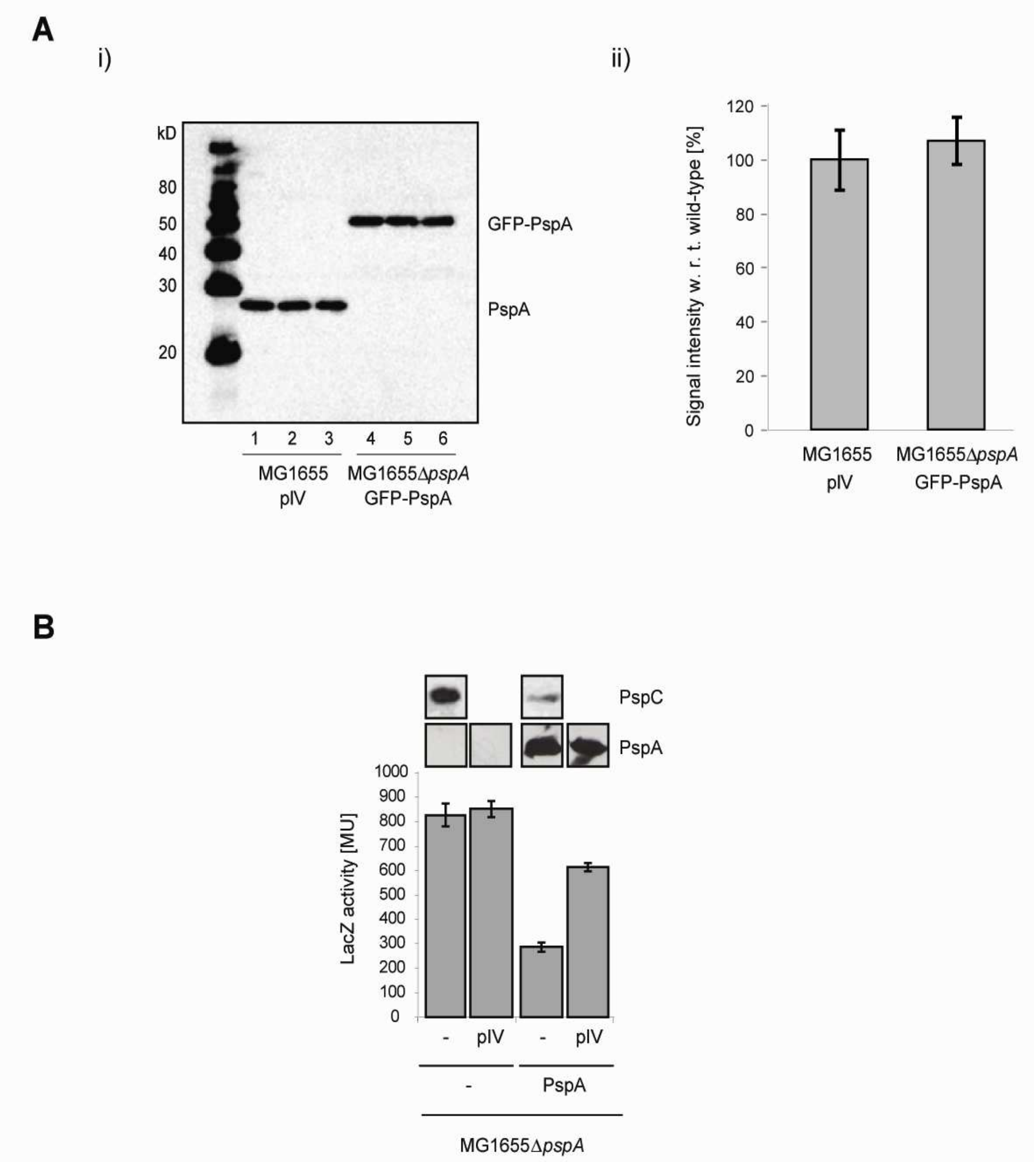

Supplementary Figure 2

A

i)

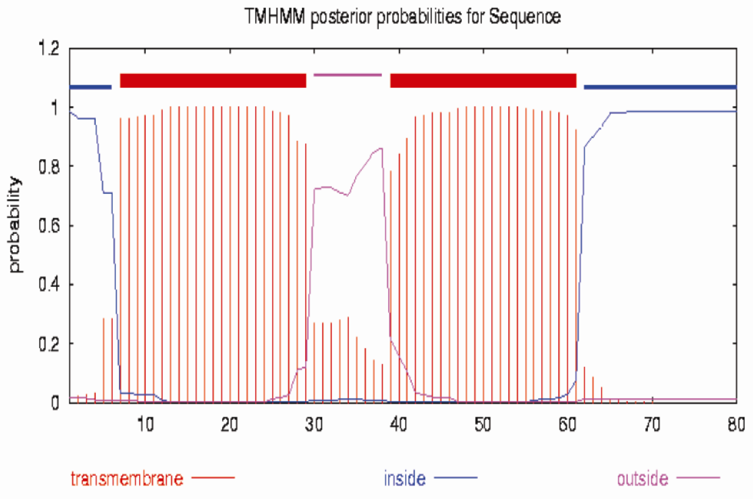

ii)

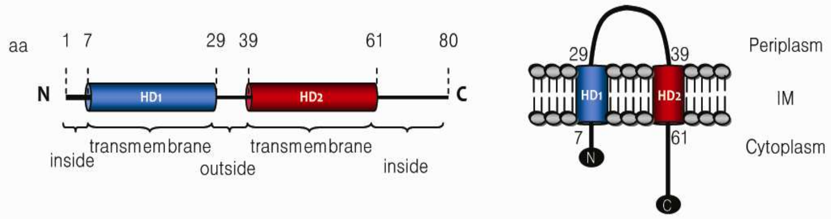

B

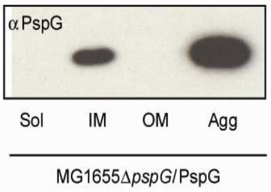

Supplementary Figure 3

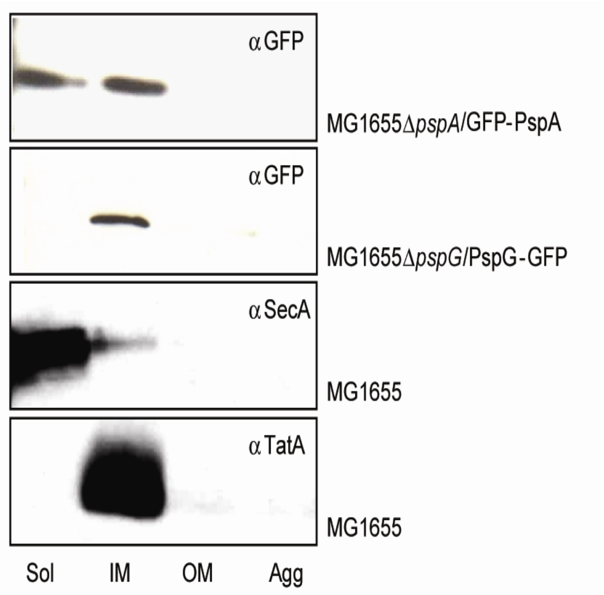

Supplementary Figure 4

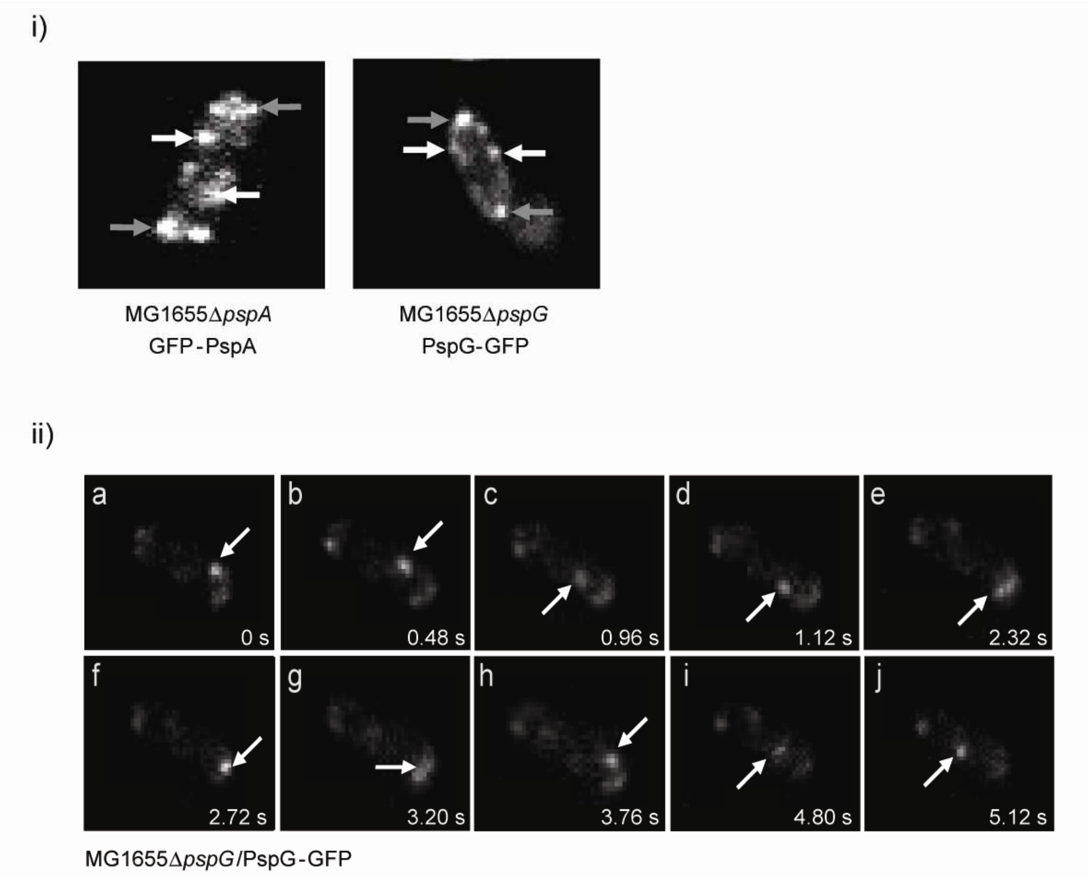

Supplementary Figure 5

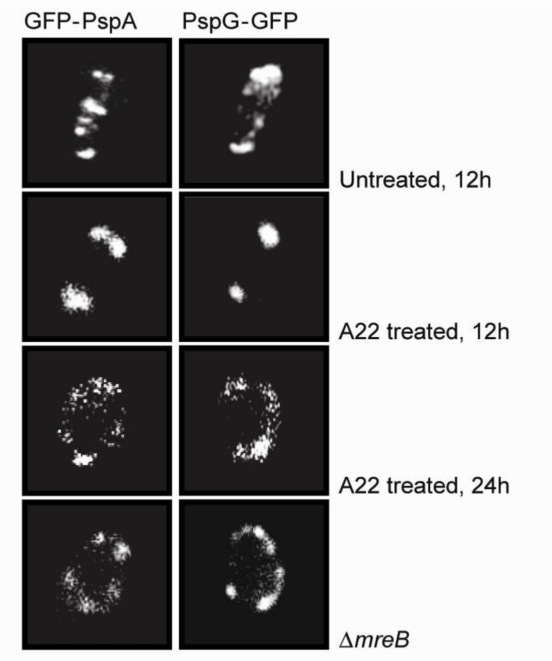

Supplementary Figure 6

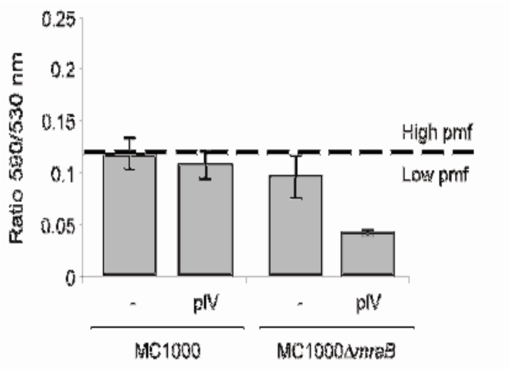

Supplementary Figure legends

<u>Supplementary Figure 1: Expression-level of GFP-PspA resembles chromosomal PspA after pIV stress.</u>

(A) i) We demonstrate that the leaky expression (as used for microscopy) of GFP-PspA (from pEC1; Lanes 4-6) was comparable to chromosomal PspA levels from MG1655 cells upon pIV-stress (Lanes 1-3). Equal numbers of cells were boiled, loaded onto a SDS-gel and subjected to immunoblotting with α-PspA antibodies. Chemi-luminescence was detected using the LAS-3000 Luminescent Image Analyzer (FUJIFILM). ii) The average luminescence intensities of the blot shown in (i) expressed as a percentage of signal intensity with respect to wild-type PspA (from MG1655 pIV). **(B)** The negative regulatory function of plasmid-borne PspA was analysed by β-galactosidase assays in MG1655Δ*pspA* cells containing a *pspA-lacZ* transcriptional reporter fusion and by immunoblotting with PspC specific antibodies, where PspA over-production causes a decrease in PspC levels (indicated by an arrow). Immunoblotting with PspA specific antibodies was used to confirm the production of PspA *in trans*.

<u>Supplementary Figure 2: PspG is an integral IM protein.</u>

(A) HMMTOP *in silico* topology analysis predicts that PspG is an integral IM protein with two trans-membrane helices between amino acid residues 7 and 31 and 40 and 61. N- and C-terminus of PspG are predicted to reside within the cytoplasm. **(B)** Triton X-100 based fractionation of *E. coli* cells containing pLL11 (*pspG* in pBAD18c under the control of an arabinose-inducible promoter). Over-expression of

wild-type PspG was induced by 0.4% arabinose. PspG was detected by immunoblotting with α-PspG antibodies and found in the IM (Lane 2) and inclusion body (Lane 4) fractions. Sol: soluble protein fraction; IM: IM protein fraction; OM: OM protein fraction; Agg: aggregated proteins (inclusion bodies).

Supplementary Figure 3: Validation of the Triton X-100 fractionation method.

The Triton X-100 fractionation method was validated using SecA and TatA in MG1655 cells as marker proteins. Using αSecA and αTatA antibodies, SecA (which is cycling between the cytoplasm and IM) was found in the soluble and IM protein fraction whereas TatA (which is an integral IM protein) was only detected in the IM protein fraction. Shown are also the locations of GFP-PspA in MG1655Δ*pspA* and of PspG-GFP in MG1655Δ*pspG*. Sol (soluble fraction; cytoplasmic and periplasmic proteins), IM (inner membrane), OM (outer membrane) and Agg (aggregated) proteins (inclusion bodies).

Supplementary Figure 4: Original epi-fluorescence images of Figure 3.

Shown are the original epi-fluorescence images of *E. coli* MG1655Δ*pspA*/GFP-PspA and MG1655Δ*pspG*/PspG-GFP cells. Polar GFP fusion proteins are indicated with grey arrows, lateral complexes with white arrows. (a-j) Original time-lapsed epi-fluorescence images of MG1655Δ*pspG*/PspG-GFP cells. The movement of one of the mobile lateral complexes is indicated in each image by a black arrow. All images were taken using a Nikon TE-2000 inverted optical microscope.

Supplementary Figure 5: Original epi-fluorescence images of Figure 4.

Shown are the original epi-fluorescence images of *E. coli* MG1655Δ*pspA*/GFP-PspA and MG1655Δ*pspG*/PspG-GFP cells before and after A22 treatment and of *E. coli* MC1000Δ*mreB* cells expressing either GFP-PspA or PspG-GFP. Either disruption or removal of MreB results in only polar GFP-PspA and PspG-GFP complexes being visible. All images were taken using Nikon TE-2000 inverted optical microscope.

Supplementary Figure 6: Pmf measurement in cells deleted for *mreB*.

Maintenance of the pmf in un-stressed (- pIV) and stressed (+ pIV) cells deleted for *mreB* was tested using the electron potential indicating dye, JC1 (Jovanovic *et al.*, 2006). Pmf was measured as the ratio between 530nm (green) and 590nm (red) averaged across 3 different microscopic fields. An increase in the ratio values corresponds to a decrease in electron potential.

Supplementary video clip: Curved and linear motions of PspG-GFP.

The video clip shows the curved and linear motions of PspG-GFP (as shown in Figure 3 ii). The video was recorded using a Nikon TE-2000 inverted optical microscope with a time-resolution of 80 ms / frame.

Supplementary References

Jones SE, Lloyd LJ, Tan KK, Buck M (2003) Secretion defects that activate the phage shock response of Escherichia coli. *J Bacteriol* **185**: 6707-6711

Karimova G, Pidoux J, Ullmann A, Ladant D (1998) A bacterial two-hybrid system based on a reconstituted signal transduction pathway. *Proc Natl Acad Sci U S A* **95**: 5752-5756

Lloyd LJ, Jones SE, Jovanovic G, Gyaneshwar P, Rolfe MD, Thompson A, Hinton JC, Buck M (2004) Identification of a new member of the phage shock protein response in Escherichia coli, the phage shock protein G (PspG). *J Biol Chem* **279**: 55707-55714

Weiss DS, Chen JC, Ghigo JM, Boyd D, Beckwith J (1999) Localization of FtsI (PBP3) to the septal ring requires its membrane anchor, the Z ring, FtsA, FtsQ, and FtsL. *J Bacteriol* **181**: 508-520
